# Sports nutrition supplements and adverse events – a meta-epidemiological study of case reports specifically addressing causality assessment

**DOI:** 10.1007/s00228-021-03223-9

**Published:** 2021-10-02

**Authors:** Rickard Zeijlon, Victor Hantelius, Susanna M. Wallerstedt, Lina Holmqvist

**Affiliations:** 1 Department of Internal Medicine, Sahlgrenska University Hospital, Gothenburg, Sweden; 2 Department of Medicine, Sahlgrenska Academy, University of Gothenburg, Gothenburg, Sweden; 3 Department of Pharmacology, Sahlgrenska Academy, University of Gothenburg, Gothenburg, Sweden; 4 HTA-Centrum, Sahlgrenska University Hospital, Gothenburg, Sweden; 5 Gothenburg Emergency Medicine Research Group, GEMREG, Sahlgrenska University Hospital, Gothenburg, Sweden

**Keywords:** Dietary supplement, Causality assessment, Clinical reasoning, Adverse event

## Abstract

**Purpose:**

This meta-epidemiological study aimed to systematically review case reports regarding sports nutrition supplements and adverse events (AEs), specifically addressing the issue of causality assessments.

**Methods:**

Through a systematic literature search we identified all published case reports of AEs associated with sports nutrition supplements between 1 January 2008 and 1 March 2019. Data regarding AEs, suspected supplements, relevant causality assessment factors and the reporting of clinical reasoning and/or systematic causality assessment methods were extracted.

**Results:**

In all, 72 publications were included, reporting 134 supplements and 37 different AEs in 97 patients (85% males; median age: 30 years [range: 14–60]). Information regarding previous health and regular prescription drugs was not presented in 30% (29/97) and 46% (45/97) of cases, respectively. In 23% (22/97) of the cases, no alternative cause was mentioned. Clinical reasoning was identified in 63% (61/97), and in 13% (8/61) of these, a systematic causality assessment method was applied. In cases with clinical reasoning, a theoretic rationale (92% vs 78%, P = 0.05), a description of previous cases (90% vs 72%, P = 0.021) and body fluid analysis (18% vs 3%, P = 0.027) were reported to a greater extent. Among cases with clinical reasoning, the application of a systematic causality assessment method captured additional important aspects: use of medication (100% vs 55%, P = 0.015), alcohol use (88% vs 43%, P = 0.020) and illicit drug use (88% vs 40%, P = 0.011).

**Conclusions:**

In published case reports where sports nutrition supplements were suspected to have caused AEs, essential factors for causality assessment were left out in a non-negligible proportion. Clinical reasoning was identified in most cases whereas a systematic causality assessment method was applied in a minority. Factors of importance for causality assessment were reported to a greater extent in cases including clinical reasoning, and the application of a systematic causality assessment method captured additional aspects of importance.

**Supplementary information:**

The online version contains supplementary material available at 10.1007/s00228-021-03223-9.

## Introduction

The use of sports nutrition supplements, to improve performance or results in sports or physical fitness [1], is extensive [2] and has been linked with adverse events (AEs). Over the last decade, several case reports have been published linking sports nutrition supplements to severe AEs, for example involving the stimulant substances Ephedra, 1,3-dimethylamylamine (DMAA) and beta-methylphenylethylamine (BMPEA) [1, 3, 4]. Dietary supplements were estimated to contribute to 23,000 emergency department visits every year in the United States (US), a figure which may be an underestimation [5, 6]. Sports nutrition supplements in turn, have been estimated to constitute 13.8% of all dietary supplements [1].

Both in the US and the European Union (EU), the marketing company is responsible for safety issues related to dietary supplements including sports nutrition supplements [7–9]. In contrast to pharmaceutical drugs which are subjected to an elaborate approval process before market access, post-market surveillance is the main source of information regarding safety for supplements, primarily through spontaneous reporting of AEs and published case reports. For manufacturers in the US, reporting of AEs to the Food and Drug Administration (FDA) is mandatory only for those defined as serious [10]. In the EU, there are no harmonized post-market regulations regarding the reporting of AEs for dietary supplements; national legislation applies with no requirement of reporting to the European Food Safety Authority (EFSA) or any other authority [11].

Spontaneous reports, from health care, consumers and dietary supplement manufacturers, have limitations and often lack information required to assess causality [12–14]. It is not known to what extent such essential information is included in published case reports where a sports nutrition supplement has been associated with an AE. In addition, we have not found any studies reporting how causality is assessed and reported in published case reports; and to what extent systematic causality assessment methods are used. Such methods are essential for signal detection and evaluation in pharmacovigilance based on spontaneous reporting of AEs. Indeed, there are several systematic methods available, none being universally accepted [15]. Causality can also be assessment through ad hoc clinical investigation of alternative causes of an AE without using a systematic causality assessment method. The term *clinical reasoning* has previously been described for this type of causality assessment of drug-related AEs, and relates to the process of clinically evaluating the probability of causality, and the exclusion of alternative causes by the means of diagnostic tools [16]. The term clinical reasoning per se is defined according to Taber’s Cyclopedic Medical Dictionary as “The use of a patient’s history, physical signs, symptoms, laboratory data, and radiological images to arrive at a diagnosis and formulate plan for treatment” [17].

The aim of this meta-epidemiological study was to review published case reports regarding sports nutrition supplements and AEs, focusing on the presented data, causality assessment through clinical reasoning and the application of systematic causality assessment methods.

## Materials and methods

To investigate the reporting patterns and the use of clinical reasoning and/or systematic causality assessment methods, we first conducted a systematic literature search in Cochrane, Embase and PubMed to find all published case reports, written in English and published between 1 January 2008 and 1 March 2019, where a sports nutrition supplement had been associated with an AE. The search was defined in a PICO (Patients, Intervention, Comparison, Outcome) statement. Patients (P) were individuals at any age; intervention (I) was sports nutrition supplements; comparison (C) was not applicable as this review focused on case reports; and Outcome (O) was any AE. Search strategies are provided in Online Resource 1.

Identified abstracts were screened by one author (R.Z.), and those that did not meet the PICO criteria were excluded. In case of uncertainties, the abstract was assessed by an additional author, followed by a consensus decision. If there were still uncertainties, the full-text article was retrieved and independently assessed by two authors, followed by a consensus discussion and a joint decision on inclusion/exclusion. For publications excluded after full-text reading, reason for exclusion was recorded. Data from the included studies were independently extracted by two authors, and potential discrepancies resolved in consensus. Data included age and sex of the patient, the sports nutrition supplement/s/ at issue, and the suspected AE/s/. We also recorded whether clinical reasoning was presented, i.e. an any investigation of alternative causes of the AE other than the sports nutrition supplement. Clinical reasoning was defined as ≥ 1 differential diagnosis mentioned and excluded by means of diagnostic measures and did not require any other specific reported factors. To further investigate the causality assessment in case reports with clinical reasoning, we recorded whether a systematic causality assessment method had been applied or not, defined as any specific method referred to by the case report authors.

To investigate the reporting of patient characteristics of relevance for causality assessment, we recorded previous health condition, as well as the use of medications, alcohol, tobacco smoking, illicit drugs and other supplements. We also recorded the reporting of AE and sports nutrition supplement characteristics of relevance for the causality assessment, including the time relationship between intake and reaction; the response to withdrawal and re-challenge; previous cases; a potential theoretic rationale; body fluid and supplement analysis and the presence of adulteration.

### Statistics

Descriptive analyses were performed using SPSS (IBM) software version 26. We used the Chi Square test to compare the reporting of characteristics of importance for the causality assessment according to the presentation of clinical reasoning (yes or no). In the subgroup of case reports that presented clinical reasoning, the same comparisons were made according to the application of a systematic causality assessment method (yes or no). The significance level was set at an alpha of 0.05.

## Results

**Fig. 1:**
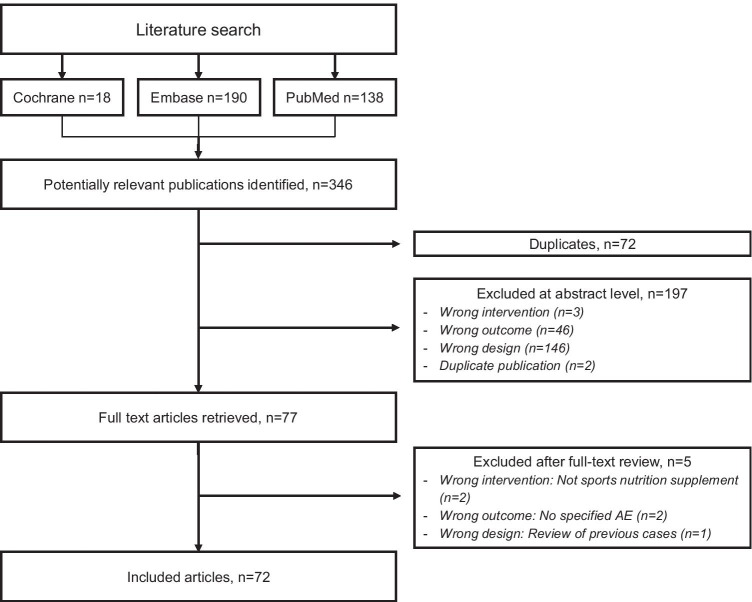
Flowchart of the study selection

After removal of duplicates, the literature search identified 277 unique publications, 72 of which fulfilled our PICO and were included in the review (Fig. 1, Online Resource 2). Exclusions after full-text reading are described in Online Resource 3. The included case reports concerned 97 patients (82 male, 13 female, 2 not specified), with a median age of 30 years (range: 14–60). Characteristics are described in Table 1.

**Table 1:** Characteristics of reported cases (n = 97)

	Prevalence in study cohort	Information provided
	n (%) if not stated otherwise	n (%) of total
Age, median (range)	30 (14–60)	96 (99%)
Male sex	82 (85%)	95 (98%)
Previously healthy*	58 (85%)	68 (70%)
Somatic disease†	8 (12%)	68 (70%)
Psychiatric disorder§	4 (6%)	68 (70%)
> 1 supplement identified as responsible for AE	20 (21%)	94 (97%)
Use of concomitant supplements**	20 (28%)	72 (74%)
Used ≥ 1 regular prescription drug/s	14 (27%)	52 (54%)
Smoked tobacco	8 (26%)	31 (32%)
Consumed alcohol	19 (49%)	39 (40%)
Used illicit drugs	3 (8%)	40 (41%)
Symptom duration < 30 days	37 (44%)	84 (87%)
Fatal AE	6 (6%)	97 (100%)
Adulteration	2 (2%)	N/A

*No chronic or regularly medicated somatic or psychiatric disease; †Any chronic somatic disease or somatic disease requiring regular prescription drug/s; §Any chronic psychiatric disorder or psychiatric disorder requiring regular prescription drug/s/; **Concomitant use of any other supplement than the one identified as responsible of adverse event

*AE *adverse event, *N/A* not applicable without analysis of sports nutrition supplement

A total of 134 different sports nutrition supplements were suspected to have caused 37 different AEs. The supplements consisted of pre-workout supplements (PWO) (n = 35), unspecified blends (n = 27), proteins (n = 22), anabolic steroids (n = 21), creatine (n = 8), hormones (n = 7), amino acids (n = 6), herbals (n = 4), unspecified energy drinks (n = 2) or were not categorizable (n = 2). The supplements were analyzed in four cases and adulteration was demonstrated in two of these (BMPEA adulteration and arsenic contamination). The most frequently reported AEs were hepatotoxicity (n = 14), acne vulgaris (n = 10), rhabdomyolysis (n = 9), acute renal injury (n = 7), acute myocardial infarction (n = 6), cardiac arrest (n = 6), acute psychosis (n = 5), and cerebral hemorrhage (n = 5). The suspected substances for the most frequently reported adverse events are presented in Table 2, and fatal cases in Table 3.

**Table 2:** Suspected substances for the most frequently reported adverse events For each AE, the number of *individuals* where the specific substance was *the only suspected substance* is provided. The number of additional *times* when the specific substance was reported as *one of the suspected substances* is provided within parentheses. For specific substances, numbers > 0 are bolded

**Substance**	Hepatotoxicity	Acne vulgaris	Rhabdo-myolysis	Acute renal injury	AMI	Cardiac arrest	Acute psychosis	Cerebral hemorrhage	Other AE*	**Total within substance**
Protein	0 (**+ 1**)	**10** (+ 0)	0 (+ 0)	0 (**+ 1**)	0 (**+ 1**)	0 (+ 0)	0 (**+ 1**)	0 (+ 0)	1	11
DMAA	0 (+ 0)	0 (+ 0)	0 (+ 0)	0 (+ 0)	0 (**+ 1**)	**2** (**+ 2**)	0 (+ 0)	**3** (**+ 1**)	1	6
Caffeine	0 (+ 0)	0 (+ 0)	**3** (**+ 2**)	0 (**+ 1**)	0 (**+ 1**)	**1** (**+ 2**)	0 (**+ 1**)	0 (**+ 1**)	0	4
Creatine	0 (**+ 1**)	0 (+ 0)	0 (+ 0)	**1** (**+ 5**)	0 (+ 0)	0 (+ 0)	0 (**+ 1**)	0 (+ 0)	2	3
Methasteron	**3** (+ 0)	0 (+ 0)	0 (+ 0)	0 (+ 0)	0 (+ 0)	0 (+ 0)	0 (+ 0)	0 (+ 0)	0	3
Testosterone	**2** (+ 0)	0 (+ 0)	0 (+ 0)	0 (**+ 4**)	0 (+ 0)	0 (+ 0)	0 (+ 0)	0 (+ 0)	1	3
Anabolic steroid	**2** (**+ 1**)	0 (+ 0)	0 (+ 0)	0 (+ 0)	0 (+ 0)	0 (+ 0)	0 (+ 0)	0 (+ 0)	1	3
Synephrine	0 (+ 0)	0 (+ 0)	0 (+ 0)	0 (+ 0)	**2** (**+ 1**)	0 (+ 0)	0 (+ 0)	0 (+ 0)	0	2
Methylstenbolone	0 (**+ 2**)	0 (+ 0)	0 (+ 0)	0 (+ 0)	0 (+ 0)	0 (+ 0)	0 (+ 0)	0 (+ 0)	0	0
Nandrolone	0 (+ 0)	0 (+ 0)	0 (+ 0)	0 (**+ 4**)	0 (+ 0)	0 (+ 0)	0 (+ 0)	0 (+ 0)	0	0
Citrus aurantium	0 (+ 0)	0 (+ 0)	0 (+ 0)	0 (+ 0)	0 (**+ 1**)	0 (+ 0)	0 (**+ 1**)	0 (+ 0)	0	0
Unknown substance	**1** (+ 0)	0 (+ 0)	**3** (+ 0)	0 (+ 0)	0 (+ 0)	**1** (+ 0)	0 (+ 0)	0 (+ 0)	3	8
Other substance†	6	0	3	6	4	2	5	2	26	54
**Total within AE**	14	10	9	7	6	6	5	5	35	97

*Other AE than those specified with either one suspected AE per individual or combinations of suspected AEs per individual; †Other substance than those specified either as one suspected substance per individual or as a combination of substances per individual

*AE* adverse event, *AMI* acute myocardial infarction, *DMAA* 1,3-dimethylamylamine

In 61 (63%) cases, clinical reasoning was presented. In the remaining 36 (37%) cases, no alternative cause of the AE/s/ other than the sports nutrition supplement was mentioned (n = 22), or an alternative cause was mentioned but without clinical reasoning (n = 14). In five of the six fatal cases, clinical reasoning was presented. When comparing cases with versus without clinical reasoning, it was more common among the former to present alcohol use, information regarding previous cases and body fluid analysis (Table 4). Analyses of body fluid was performed in five of the six fatal cases. In cases with clinical reasoning, there was also a trend towards more frequent reporting of a potential theoretic rationale and concurrent use of medications and/or other supplements.

**Table 3:** Fatal cases

Case No	Year	Age	Sex	Co-morbidity	Substance	Supplement name	Adverse event	Cause of death
1 [39]	2010	42	Male	Diabetes	Creatine (in combination with metformin)	Not specified	Acute renal failure with lactate acidosis	Cardiac arrest
2 [40]	2012	32	Male	Sickle cell trait	DMAA, caffeine	Not specified	Cardiac arrest	Cardiac arrest
3 [40]	2012	22	Female	Previously healthy	DMAA, caffeine	Not specified	Cardiac arrest	Cardiac arrest
4 [41]	2013	35	Male	Previously healthy	Arsenic, anabolic steroid (arsenic contamination found in supplement analysis)*	Performance enhancer (unspecified)	Arsenic poisoning	Multi-organ failure
5 [42]	2013	39	Male	NR	Caffeine	Caffeine Anhydrous Powder	Cardiac arrest	Cardiac arrest
6 [43]	2015	30	Female	Previously healthy	DMAA	Jack3D	Cardiac arrest	Cardiac arrest

*Substances originating from > 1 supplement product

*DMAA* 1,3-dimethylamylamine, *NR* not reported

In 8 (13%) cases with clinical reasoning, a systematic causality assessment method was applied. The Naranjo Adverse Drug Reaction Probability scale [18] was used in 3 cases [19–21], the CIOMS/RUCAM (Council for International Organizations of Medical Sciences/Roussel Uclaf Causality Assessment Method) [22, 23] in 2 cases [24, 25], the WHO/UMC (World Health Organization-Uppsala Monitoring Center) causality assessment system [26] in 1 case [4], and the Teschke scale [27] in 1 case [28]. In 1 case [29], both the WHO/UMC causality assessment system and CIOMS/RUCAM were used. In none of the six fatal cases, a systematic causality assessment method was applied Among the case reports using clinical reasoning to support a relationship between the sports nutrition supplement and the AE, it was more common to report use of medication, alcohol consumption and illicit drug use when a systematic causality assessment method was applied, with a trend towards presenting previous health more often (Table 5). There was also a trend towards using systematic causality assessment methods more often for hepatopancreatic AEs compared to AEs from other organ systems (n = 4 [50%] versus n = 11 [21%], P = 0.073).

**Table 4:** Causality assessments factors, according to the use of clinical reasoning

Factors of relevance for causality assessment			Clinical reasoning		
		All (n=97)	Yes (n=61)	No (n=36)	P-value
Information reported regarding…					
Patient characteristics	Previous health condition	68 (67%)	44 (72%)	24 (67%)	0.57
	Use of medications	52 (54%)	37 (61%)	15 (42%)	0.070
	Smoking status	31 (32%)	18 (30%)	13 (36%)	0.50
	Alcohol use	39 (40%)	30 (49%)	9 (25%)	0.019
	Illicit drug use	40 (41%)	28 (46%)	12 (33%)	0.22
	Concomitant supplement use*	72 (74%)	49 (80%)	23 (64%)	0.074
AE characteristics	Positive time relationship	95 (98%)	59 (97%)	36 (100%)	0.27
	Response to withdrawal	78 (80%)	49 (80%)	29 (81%)	0.98
	Previous cases	81 (84%)	55 (90%)	26 (72%)	0.021
	Re-challenge†	2 (2%)	1 (2%)	1 (3%)	0.70
Supplement characteristics	Theoretic rationale	84 (87%)	56 (92%)	28 (78%)	0.050
	Body fluid analysis**	12 (12%)	11 (18%)	1 (3%)	0.027
	Supplement analysis††	4 (4%)	3 (5%)	1 (3%)	0.61

*Concomitant use of any other supplement than the one identified as responsible of adverse event; †Culprit supplement or substance re-administered for the purpose of reproducing observed index adverse event; **In vivo analysis of substances from sports nutrition supplement in body fluid; ††In vitro analysis of the sports nutrition supplement

*AE* adverse event

**Table 5:** Causality assessments factors, according to the use systematic causality assessment methods

Factors of relevance for causality assessment			Systematic causality assessment		
		All* (n=61)	Yes (n=8)	No (n=53)	P-value
Information reported regarding…					
Patient characteristics	Use of medications	68 (70%)	8 (100%)	36 (68%)	0.059
	Smoking status	37 (61%)	8 (100%)	29 (55%)	0.015
	Alcohol use	18 (30%)	4 (50%)	14 (26%)	0.17
	Illicit drug use	30 (49%)	7 (88%)	23 (43%)	0.020
	Concomitant supplement use†	28 (46%)	7 (88%)	21 (40%)	0.011
	Use of medications	49 (80%)	7 (88%)	42 (79%)	0.58
AE characteristics	Positive time relationship	59 (97%)	8 (100%)	51 (96%)	0.58
	Response to withdrawal	49 (80%)	8 (100%)	41 (77%)	0.13
	Previous cases	55 (90%)	7 (88%)	48 (91%)	0.79
	Re-challenge**	1 (2%)	0 (0%)	1 (2%)	0.70
Sports nutrition supplement characteristics	Theoretic rationale	56 (92%)	7 (88%)	49 (93%)	0.63
	Body fluid analysis††	11 (18%)	0 (0%)	11 (21%)	0.16
	Supplement analysis§	3 (5%)	1 (13%)	2 (4%)	0.29

^*^All cases with clinical reasoning; †Concomitant use of any other supplement than the one identified as responsible of adverse event; **Culprit supplement or substance re-administered for the purpose of reproducing observed index adverse event; ††In vivo analysis of substances from sports nutrition supplement in body fluid; §In vitro analysis of the sports nutrition supplement

*AE* adverse event

## Discussion

Three out of ten published cases, where a sports nutrition supplement had been suspected to have caused an AE, lacked information regarding previous health of the affected individual. In addition, almost half of the cases lacked information regarding the use of regular prescription drugs. In almost two thirds of the cases, clinical reasoning was reported, that is, the negation of alternative causes by means of investigations. However, in about one fifth, an alternative cause of the AE was not mentioned at all. Our findings illustrate that case reports of events suspected to be related to sports nutrition supplements could be improved, to increase their value for surveillance and signal detection. Clinical reasoning could be presented, and systematic causality assessment methods could be applied, to a greater extent.

In cases including clinical reasoning, several factors of relevance for the causality assessment were reported to a greater extent compared with cases without such reasoning. It is important to note that our definition of clinical reasoning was entirely independent of the specific factors presented as relevant for causality assessment. Clinical reasoning captured a traditional approach to assessing causality when any substance is suspected to have caused a reaction or disease, whereas the factors of relevance for causality assessment were specific and pre-defined variables. Among the 61 cases with clinical reasoning, a systematic causality assessment method was applied in 8 cases. With the use of such a systematic method, several additional factors relevant for the causality assessment were reported to an increased extent. Further, most factors relevant for a causality assessment were numerically higher in cases where a systematic causality assessment had been applied, although not reaching statistical significance. Lack of power may be an issue in these cases, and a larger sample size would have been needed to elucidate these differences further. That said, our results indicate that the use of a systematic causality assessments may be superior to not using such a method, and may be superior to traditional ad hoc differential diagnostics.

The most common systematic causality assessment method in our review was the Naranjo score. The literature is scarce regarding the use of such methods to assess AEs suspected for dietary supplements. One study compared the agreement between different assessors when analyzing spontaneous reports from consumers using modified versions of the Naranjo score and an algorithm from the FDA, with more than substantial agreement, however adding little information about the validity of the causality assessments per se [30].

In our study, there was a trend towards an increased use of systematic causality assessment methods in case reports of hepatopancreatic AEs compared to AEs from other organ systems. This finding could be attributable to the fact that the CIOMS/RUCAM-score was initially developed to assess if liver damage was associated with certain pharmaceutical drugs [15], and the Naranjo score, although developed for all drug reactions, is widely used for drug-induced liver injury [18, 31–33].

We found a substantial variation in both the severity of AEs and the organ systems being affected. It is important to note that the summarized AEs presented in this study does not reflect the risks associated with sports nutrition supplements, but only the most *reported* AEs. We also found a wide variety of sports nutrition supplements being suspected to have caused an AE. For many AEs, more than one substance in a single supplement, or multiple supplements in combination, were suspected. Since the theoretic rationale supporting an association between a supplement and an AE is mainly applicable at the substance level, the specific substances may be more important than the specific supplement. Although our compilation of reported cases does not reflect the hazard of certain substances, it can serve as a basis for physicians when an AE is suspected, potentially contributing to relevant investigations in the specific patient.

An aspect that complicates causality assessments between a sports nutrition supplement and a suspected AE is the risk of adulteration, i.e. the “spiking” of products with unlabeled synthetic substances [4, 34]. In our study, adulteration was identified in two cases. However, the supplements were analyzed in only four cases, leaving 93 uninvestigated cases. Finally, it is important to point out that the reported fatal cases do not reflect the mortality associated with sports nutrition supplements, but merely describes fatal cases that have been reported, including the specific details.

Published case reports hold advantages compared with spontaneous reports. Although this review shows that there may be room for improvements regarding the reporting of issues of importance for causality assessments, detailed information is often available regarding why causality was suspected, investigations made, and how the AE was managed and treated. This may partly be attributed to the fact that the authors often are the treating physicians [12, 35, 36]. In spontaneous reports, on the other hand, available information, sometimes provided by health professionals and sometimes by the public, rarely suffices to determine more than a possible causal relationship [37], implying that the event could just as well have been caused by an emerging or worsening disease. Other strengths with published case reports are that these articles undergo peer review and are accessible through e.g. PubMed. They also often include reviews of previous cases which provides educational insights which can trigger others to report similar events [35].

A thorough investigation or argumentation of alternative causes other than the sports nutrition supplement is essential; confirmation bias and the inability to validate substances related to the AEs are often put forward as main limitations of case reports in general [12, 13]. However, case reports can generate hypotheses that can be further evaluated. In a study of 83 drug withdrawals due to published case reports of potentially fatal drug-related AEs, confirmatory studies had been conducted in 57 cases where evidence of an association was found in 52 [38]. Though generally ranked lowest on the evidentiary hierarchy, published case reports play a vital role in drug safety. Indeed, given the limited availability of evidence regarding effects of sports nutrition supplements, such reports may constitute the main source for signal detection.

In the present review, the value of systematic causality assessment methods as add-on to clinical reasoning was investigated. With the inverse approach, i.e., clinical reasoning as add-on to systematic causality assessment methods, some patient/substance characteristics appear that would perhaps not be captured if the latter methods are used alone. Indeed, smoking is not included in any of the systematic tools used in the studied case reports. Further, use of illicit drugs is included only in the Teschke scale [27]. Interestingly, none of the systematic causality assessment tools include an analysis of the contents of the product. Consequently, adulteration would not be captured if strictly following the protocol. As smoking, use of illicit drugs, and adulteration are important factors when assessing causality between a sports nutrition supplement and an event, inclusion of these aspects in standardized tools could be considered. The main strengths of this meta-epidemiological study are the comprehensive data collection; the systematic literature search and study selection; and the novel aspect of investigating causality assessment for AEs related to sports nutrition supplements. The small number of case reports where a systematic causality assessment had been made is, however, a limitation, with a risk of statistical type II errors. Consequently, non-significant differences between these groups have to be interpreted with caution.

In conclusion, a non-negligible proportion of published case reports, where sports nutrition supplements have been suspected to have caused AEs, leave out essential factors for the causality assessment. Clinical reasoning could be identified in most cases but a systematic causality assessment method was applied in a minority. Factors relevant for causality assessment were provided to a greater extent in cases with clinical reasoning. Among these, case reports applying a systematic causality assessment method were less likely to leave out additional such information. An increased use of systematic causality tools in future case reports may increase their value for the surveillance of these supplements.

## Supplementary information

Below is the link to the electronic supplementary material.

Supplementary file1 (DOCX 24 KB)

Supplementary file2 (DOCX 24 KB)

Supplementary file3 (DOCX 18 KB)

## Data Availability

The datasets generated during and/or analyzed during the current study are available from the corresponding author on reasonable request.
