# Supplementary material for: Sports nutrition supplements and adverse events – a meta-epidemiological study of case reports specifically addressing causality assessment
Source: Eur J Clin Pharmacol. 2021 Oct 2;78(1):1–9. doi: 10.1007/s00228-021-03223-9 (PMC8724217; doi:10.1007/s00228-021-03223-9)
Supplement: Supplementary file 1 — Supplementary file1 (DOCX 24 KB) [file 228_2021_3223_MOESM1_ESM.docx]

**Sports nutrition supplements and adverse events – a meta-epidemiological study of case reports specifically addressing causality assessment**

*Journal name: European Journal of Clinical Pharmacology*

Authors: Rickard Zeijlon, MD^1,2^, Victor Hantelius, MD^1^, Susanna M. Wallerstedt, MD, Professor^3,4^, Lina Holmqvist, MD, PhD^1,2^

^1^Department of Internal Medicine, Sahlgrenska University Hospital/S, Gothenburg, Sweden

^2^Department of Medicine, Sahlgrenska Academy, University of Gothenburg, Sweden

^3^Department of Pharmacology, Sahlgrenska Academy, University of Gothenburg, Sweden

^4^HTA Centre, Sahlgrenska University Hospital, Gothenburg, Sweden

E-mail of corresponding author:

rickard.zeijlon@gu.se

Online Resource 1. Literature search strategies

| Database: PubMed; date: 2019-03-19; no of results: 138 ref. | | |
| --- | --- | --- |
| Search | Query | Items found |
| [**#14**](https://www.ncbi.nlm.nih.gov/pubmed/advanced) | **Search #7 NOT #8 Filters: published in the last 10 years; Swedish; Norwegian; English; Danish** | [**138**](https://www.ncbi.nlm.nih.gov/pubmed/?cmd=HistorySearch&querykey=14) |
| [#13](https://www.ncbi.nlm.nih.gov/pubmed/advanced) | Search #7 NOT #8 Filters: published in the last 10 years; Swedish; Norwegian; English | [138](https://www.ncbi.nlm.nih.gov/pubmed/?cmd=HistorySearch&querykey=13) |
| [#12](https://www.ncbi.nlm.nih.gov/pubmed/advanced) | Search #7 NOT #8 Filters: published in the last 10 years; Swedish; Norwegian | [1](https://www.ncbi.nlm.nih.gov/pubmed/?cmd=HistorySearch&querykey=12) |
| [#11](https://www.ncbi.nlm.nih.gov/pubmed/advanced) | Search #7 NOT #8 Filters: published in the last 10 years; Swedish | [1](https://www.ncbi.nlm.nih.gov/pubmed/?cmd=HistorySearch&querykey=11) |
| [#10](https://www.ncbi.nlm.nih.gov/pubmed/advanced) | Search #7 NOT #8 Filters: published in the last 10 years | [140](https://www.ncbi.nlm.nih.gov/pubmed/?cmd=HistorySearch&querykey=10) |
| [#9](https://www.ncbi.nlm.nih.gov/pubmed/advanced) | Search #7 NOT #8 | [214](https://www.ncbi.nlm.nih.gov/pubmed/?cmd=HistorySearch&querykey=9) |
| [#8](https://www.ncbi.nlm.nih.gov/pubmed/advanced) | Search ((animals[mh]) NOT (animals[mh] AND humans[mh])) OR animal[ti] OR animals[ti] OR rat[ti] OR rats[ti] OR mouse[ti] OR mice[ti] OR rodent[ti] OR rodents[ti] OR dog[ti] OR dogs[ti] OR cat[ti] OR cats[ti] OR hamster[ti] OR hamsters[ti] OR rabbit[ti] OR rabbits[ti] OR Swine[ti] | [4,853,687](https://www.ncbi.nlm.nih.gov/pubmed/?cmd=HistorySearch&querykey=8) |
| [#7](https://www.ncbi.nlm.nih.gov/pubmed/advanced) | Search #5 AND #6 | [219](https://www.ncbi.nlm.nih.gov/pubmed/?cmd=HistorySearch&querykey=7) |
| [#6](https://www.ncbi.nlm.nih.gov/pubmed/advanced) | Search “a case”[tiab] OR “case reports”[tiab] OR “case report”[tiab] OR case reports[pt] | [2,270,814](https://www.ncbi.nlm.nih.gov/pubmed/?cmd=HistorySearch&querykey=6) |
| [#5](https://www.ncbi.nlm.nih.gov/pubmed/advanced) | Search #3 OR #4 | [7,503](https://www.ncbi.nlm.nih.gov/pubmed/?cmd=HistorySearch&querykey=5) |
| [#4](https://www.ncbi.nlm.nih.gov/pubmed/advanced) | Search Performance-Enhancing Substances[mesh] OR performance-enhancing substance*[tiab] OR performance-enhancing supplement*[tiab] OR performance-enhancing drug*[tiab] OR pre-workout supplement*[tiab] OR pre-workout booster[tiab] OR pre-workout beverage*[tiab] OR pre-workout drink*[tiab] OR sport supplement*[tiab] OR sports supplement*[tiab] | [1,290](https://www.ncbi.nlm.nih.gov/pubmed/?cmd=HistorySearch&querykey=4) |
| [#3](https://www.ncbi.nlm.nih.gov/pubmed/advanced) | Search #1 AND #2 | [6,539](https://www.ncbi.nlm.nih.gov/pubmed/?cmd=HistorySearch&querykey=3) |
| [#2](https://www.ncbi.nlm.nih.gov/pubmed/advanced) | Search exercise[tiab] OR exercising[tiab] OR exercise[Mesh] OR physical fitness[mesh] OR physical activit*[tiab] OR training*[tiab] OR exercise training[tiab] OR workout*[tiab] OR gym[tiab] OR bodybuild*[tiab] OR weightlift*[tiab] OR fitness[tiab] OR athlet*[tiab] OR sport[tiab] OR sports[tiab] | [813,201](https://www.ncbi.nlm.nih.gov/pubmed/?cmd=HistorySearch&querykey=2) |
| [#1](https://www.ncbi.nlm.nih.gov/pubmed/advanced) | Search dietary supplements[mesh] OR dietary supplement*[tiab] OR food supplement*[tiab] OR diet supplement*[tiab] OR nutritional supplement*[tiab] OR herbal supplements[tiab] | [87,675](https://www.ncbi.nlm.nih.gov/pubmed/?cmd=HistorySearch&querykey=1) |

| Database: Embase (OVID) 1974 to 2019 March 18; date: 2019-03-19; no of results: 190 ref. | | |
| --- | --- | --- |
| # | Searches | Results |
| 1 | exp dietary supplement/ | 8,563 |
| 2 | (dietary supplement* or food supplement* or diet supplement* or nutritional supplement* or herbal supplement*).ab,ti. | 40,777 |
| 3 | 1 or 2 | 46,076 |
| 4 | exp exercise/ or exp fitness/ | 328,613 |
| 5 | (exercise or exercising or physical fitness or physical activit* or training* or exercise training or workout* or gym or bodybuild* or weightlift* or athlet* or sport or sports).ab,ti. | 911,268 |
| 6 | 4 or 5 | 998,363 |
| 7 | exp performance enhancing substance/ | 635 |
| 8 | (performance-enhancing substance* or performance-enhancing supplement* or performance-enhancing drug* or pre-workout supplement* or pre-workout booster or pre-workout beverage* or pre-workout drink* or sport supplement* or sports supplement*).ab,ti. | 677 |
| 9 | 7 or 8 | 1,104 |
| 10 | 3 and 6 | 3,985 |
| 11 | 9 or 10 | 4,927 |
| 12 | case report/ | 2,323,239 |
| 13 | ("a case" or "case reports" or "case report").ab,ti. | 939,662 |
| 14 | 12 or 13 | 2,573,612 |
| 15 | 11 and 14 | 238 |
| 16 | limit 15 to yr="2008 -Current" | 196 |
| 17 | limit 16 to (danish or english or norwegian or swedish) | 193 |
| 18 | animals/ | 1,206,952 |
| 19 | animals/ and humans/ | 269,860 |
| 20 | 18 or 19 | 1,206,952 |
| **21** | **17 not 20** | **190** |

| Database: The Cochrane Library; date: 2019-03-19; no of results: 18 ref. | | |
| --- | --- | --- |
| ID | Search | Hits |
| #1 | dietary supplements | 11,019 |
| #2 | (dietary supplement* OR food supplement* OR diet supplement* OR nutritional supplement* OR herbal supplement*):ti,ab,kw (Word variations have been searched) | 24,548 |
| #3 | #1 OR #2 | 24,790 |
| #4 | MeSH descriptor: [Exercise] explode all trees | 21,453 |
| #5 | MeSH descriptor: [Physical Fitness] explode all trees | 2,819 |
| #6 | (exercise OR exercising OR physical OR physical activit* OR training* OR exercise training OR workout* OR gym OR bodybuild* OR weightlift* OR fitness OR athlet* OR sport OR sports):ti,ab,kw (Word variations have been searched) | 160,310 |
| #7 | #4 OR #5 OR #6 | 161,289 |
| #8 | #3 AND #7 | 5,023 |
| #9 | MeSH descriptor: [Performance-Enhancing Substances] explode all trees | 140 |
| #10 | (performance-enhancing substance* OR performance-enhancing supplement* OR performance-enhancing drug* OR pre-workout supplement* OR pre-workout booster OR pre-workout beverage* OR pre-workout drink* OR sport supplement* OR sports supplement*):ti,ab,kw | 738 |
| #11 | #9 OR #10 | 738 |
| #12 | #8 OR #11 | 5,266 |
| #13 | (“a case” OR “case reports” OR “case report”):ti,ab,kw | 8,388 |
| #14 | (case reports):pt (Word variations have been searched) | 1,558 |
| #15 | #13 OR #14 | 9,796 |
| **#16** | **#12 AND #15** | **18** |
