# Supplementary material for: Sports nutrition supplements and adverse events – a meta-epidemiological study of case reports specifically addressing causality assessment
Source: Eur J Clin Pharmacol. 2021 Oct 2;78(1):1–9. doi: 10.1007/s00228-021-03223-9 (PMC8724217; doi:10.1007/s00228-021-03223-9)
Supplement: Supplementary file 2 — Supplementary file2 (DOCX 24 KB) [file 228_2021_3223_MOESM2_ESM.docx]

**Sports nutrition supplements and adverse events – a meta-epidemiological study of case reports specifically addressing causality assessment**

*Journal name: European Journal of Clinical Pharmacology*

Authors: Rickard Zeijlon, MD^1,2^, Victor Hantelius, MD^1^, Susanna M. Wallerstedt, MD, Professor^3,4^, Lina Holmqvist, MD, PhD^1,2^

^1^Department of Internal Medicine, Sahlgrenska University Hospital/S, Gothenburg, Sweden

^2^Department of Medicine, Sahlgrenska Academy, University of Gothenburg, Sweden

^3^Department of Pharmacology, Sahlgrenska Academy, University of Gothenburg, Sweden

^4^HTA Centre, Sahlgrenska University Hospital, Gothenburg, Sweden

E-mail of corresponding author:

rickard.zeijlon@gu.se

Online Resource 2. Full references for included articles.

1. Agbenyefia P, Arnold CA, Kirkpatrick R, 3rd. Cholestatic Jaundice With the Use of Methylstenbolone and Dymethazine, Designer Steroids Found in Super DMZ Rx 2.0 "Nutritional Supplement": A Case Report. Journal of investigative medicine high impact case reports. 2014;2(2):
2. Almukhtar SE, Abbas AA, Muhealdeen DN, Hughson MD. Acute kidney injury associated with androgenic steroids and nutritional supplements in bodybuilders. Clinical Kidney Journal. 2015;8(4):415-9.
3. Ampuero J, Garcia ES, Lorenzo MM, Calle R, Ferrero P, Gomez MR. Stanozolol-induced bland cholestasis. Gastroenterologia y hepatologia. 2014;37(2):71-2.
4. Archer JR, Dargan PI, Lostia AM, van der Walt J, Henderson K, Drake N, et al. Running an unknown risk: a marathon death associated with the use of 1,3-dimethylamylamine (DMAA). Drug testing and analysis. 2015;7(5):433-8.
5. Avelar-Escobar G, Mendez-Navarro J, Ortiz-Olvera NX, Castellanos G, Ramos R, Gallardo-Cabrera VE, et al. Hepatotoxicity associated with dietary energy supplements: use and abuse by young athletes. Annals of hepatology. 2012;11(4):564-9.
6. Binet Q, Dufour I, Agneessens E, Debongnie JC, Aouattah T, Covas A, et al. The second case of a young man with L-arginine-induced acute pancreatitis. Clinical journal of gastroenterology. 2018;11(5):424-7.
7. Boos CJ, White SH, Bland SA, McAllister PD. Dietary supplements and military operations: caution is advised. Journal of the Royal Army Medical Corps. 2010;156(1):41-3.
8. Brazeau MJ, Castaneda JL, Huitron SS, Wang J. A Case Report of Supplement-Induced Hepatitis in an Active Duty Service Member. Military medicine. 2015;180(7):e844-6.
9. Brett CN, Roberts SJ. Possible abnormal response to rocuronium in a patient taking multiple fitness supplements. Anaesthesia and intensive care. 2016;44(5):640-1.
10. Carol ML. Hydroxycut weight loss dietary supplements: a contributing factor in the development of exertional rhabdomyolysis in three U.S. Army soldiers. Military medicine. 2013;178(9):e1039-42.
11. Casella M, Dello Russo A, Izzo G, Pieroni M, Andreini D, Russo E, et al. Ventricular arrhythmias induced by long-term use of ephedrine in two competitive athletes. Heart and vessels. 2015;30(2):280-3.
12. Chahla E, Hammami M, Befeler A. Hepatotoxicity associated with anabolic androgenic steroids present in over-the-counter supplements: A case series. American Journal of Gastroenterology. 2014;2):S126.
13. Coffey DD. Unexplained hypoxia in an in-flight emergency. Aviation, space, and environmental medicine. 2014;85(6):662-7.
14. Cohen PA, Zeijlon R, Nardin R, Keizers PH, Venhuis B. Hemorrhagic Stroke Probably Caused by Exercise Combined With a Sports Supplement Containing beta-Methylphenyl-ethylamine (BMPEA): A Case Report. Annals of internal medicine. 2015;162(12):879-80.
15. Dehoney S, Wellein M. Rhabdomyolysis associated with the nutritional supplement Hydroxycut. American Journal of Health-System Pharmacy. 2009;66(2):142-8.
16. Detopoulou P, Papamikos V. Gastrointestinal bleeding after high intake of omega-3 fatty acids, cortisone and antibiotic therapy: a case study. International journal of sport nutrition and exercise metabolism. 2014;24(3):253-7.
17. Djordjevic S, Kitic D, Kostic M, Apostolovic B, Brankovic S, Ciric IM, et al. Joint influence of protein supplements, soft drinks and extreme physical activity on the development of acute renal failure and hypokalaemia. West Indian Medical Journal. 2017;66(2).
18. Doctorian T, Do B. Ascending aortic dissection in a young patient using a synephrine-containing workout supplement. Journal of cardiology cases. 2017;15(5):150-2.
19. El Rahi C, Thompson-Moore N, Mejia P, De Hoyos P. Successful use of N-acetylcysteine to treat severe hepatic injury caused by a dietary fitness supplement. Pharmacotherapy. 2015;35(6):e96-e101.
20. Eliason MJ, Eichner A, Cancio A, Bestervelt L, Adams BD, Deuster PA. Case reports: Death of active duty soldiers following ingestion of dietary supplements containing 1,3-dimethylamylamine (DMAA). Military medicine. 2012;177(12):1455-9.
21. Escamilla-Ocanas CE, Camara-Lemarroy CR, Cantu-Martinez L, Martinez HR. Acute toxic leukoencephalopathy associated with a non-prescription weight loss supplement: a report of two cases. Neurological Sciences. 2017;38(12):2199-201.
22. Flynn A, Lincoln J, Burke M. Homicidality and Psychosis Caused by an Over-the-Counter Performance-Enhancing Supplement Containing Dendrobium Extract and L-Dopa. P & T : a peer-reviewed journal for formulary management. 2016;41(6):381-4.
23. Ge Y, Liu S, Singh SM. Strong Man with a Weak Heart: The Ill Effects of Performance-Enhancing Drug Use. The American journal of medicine. 2017;130(1):e5-e7.
24. Gee P, Tallon C, Long N, Moore G, Boet R, Jackson S. Use of recreational drug 1,3 Dimethylamylamine (DMAA) [corrected] associated with cerebral hemorrhage. Annals of emergency medicine. 2012;60(4):431-4.
25. Goodison G, Overeem K, De Monte V, Siskind D. Mania associated with self-prescribed acetyl-L-carnitine in a man with bipolar I disorder. Australasian Psychiatry. 2017;25(1):13-4.
26. Hannabass K, Olsen KR. Fat burn X: burning more than fat. BMJ case reports. 2016;2016.
27. Harris BF, Winn C, Ableman TB. Hemorrhagic Stroke in a Young Healthy Male Following Use of Pre-Workout Supplement Animal Rage XL. Military medicine. 2017;182(9):e2030-e3.
28. Herbst E, McCaslin S, Kalapatapu RK. Use of Stimulants and Performance Enhancers During and After Trauma Exposure in a Combat Veteran: A Possible Risk Factor for Posttraumatic Stress Symptoms. The American journal of psychiatry. 2017;174(2):95-9.
29. Hoedebecke K, Rerucha C, Maxwell K, Butler J. Drug-induced liver injury secondary to testosterone prohormone dietary supplement use. Journal of special operations medicine : a peer reviewed journal for SOF medical professionals. 2013;13(4):1-5.
30. Iyer PS, Yelisetti R, Miriyala V, Siddiqui W, Kaji A. A remarkable case of rhabdomyolysis associated with ingestion of energy drink 'neon volt'. Journal of community hospital internal medicine perspectives. 2016;6(5):32528.
31. Jabbar SB, Hanly MG. Fatal caffeine overdose: a case report and review of literature. The American journal of forensic medicine and pathology. 2013;34(4):321-4.
32. Jeter J, DeZee KJ, Kennedy L. A Case of Paraspinal Muscle Rhabdomyolysis in a 22-Year-Old Male After Ingesting a Supplement Containing Higenamine. Military medicine. 2015;180(7):e847-9.
33. Karnatovskaia LV, Leoni JC, Freeman ML. Cardiac arrest in a 21-year-old man after ingestion of 1,3-DMAA-containing workout supplement. Clinical journal of sport medicine : official journal of the Canadian Academy of Sport Medicine. 2015;25(1):e23-5.
34. Kayapinar O, Ozde C, Ay EK, Keskin M, Kaya A. Anterior myocardial infarction in a 26-year-old body builder with concomitant use of whey protein powder and amino acid capsules. Acta Cardiologica Sinica. 2018;34(4):359-62.
35. Li C, Adhikari BK, Gao L, Zhang S, Liu Q, Wang Y, et al. Performance-Enhancing Drugs Abuse Caused Cardiomyopathy and Acute Hepatic Injury in a Young Bodybuilder. American journal of men's health. 2018;12(5):1700-4.
36. Li W, Madhira B. Phenibut (beta-Phenyl-gamma-Aminobutyric Acid) Psychosis. American journal of therapeutics. 2017;24(5):e639-e40.
37. Liane BJ, Magee C. Guerilla Warfare on the Pancreas? A Case of Acute Pancreatitis From a Supplement Known to Contain Anabolic-Androgenic Steroids. Military medicine. 2016;181(10):e1395-e7.
38. Losa F, Deidda M, Arcolaci A, Barca MP, Paoletti G, Solinas A, et al. Indian Ayruvedic supplement and physical activity as triggers of anaphylaxis related to mast cell activation syndrome: A case report. Allergy: European Journal of Allergy and Clinical Immunology. 2018;73 (Supplement 105):277.
39. Low A, Dovey J, Ash-Miles J. Vertebral artery dissection in weightlifter with performance enhancing drug use. BMJ case reports. 2011;2011.
40. Magee CD, Moawad FJ, Moses F. NO-Xplode: a case of supplement-associated ischemic colitis. Military medicine. 2010;175(3):202-5.
41. Mascagni P, Melandro F, Larghi Laureiro Z, Mennini G, Rossi M. Spontaneous hepatic rupture in a bodybuilder: a case report and review of the literature. Revista espanola de enfermedades digestivas : organo oficial de la Sociedad Espanola de Patologia Digestiva. 2018;110(4):254-6.
42. Mayer KN, Wyder D, Spasic D, Herren T. Severe rhinovirus pneumonia in a young woman taking performance-enhancing drugs. BMJ case reports. 2016;2016.
43. McDonald TJ, Perry MH, Jones AG, Donohoe M, Salzmann MB, O'Connor J. A novel case of a raised testosterone and LH in a young man. Clinica Chimica Acta. 2011;412(21-22):1999-2001.
44. Milton RM, Kelly-Rehm MC, Brahm NC, Fox MD. Hypertension in an adolescent secondary to performance enhancement supplement use. Journal of Pharmacy Practice. 2013;26 (3):344.
45. Minervini L, Antonielli Romanini F, Solmi M, Passamani A, Sferrazza E, Schifano F. Acute psychotic episode associated with the intake of a testosterone-enhancer herbal mixture purchased online. Psychotherapy and psychosomatics. 2012;81(4):248-9.
46. Parr MK, Koehler K, Geyer H, Guddat S, Schanzer W. Clenbuterol marketed as dietary supplement. Biomedical Chromatography. 2008;22(3):298-300.
47. Patel YA, Marzella N. Dietary Supplement-Drug Interaction-Induced Serotonin Syndrome Progressing to Acute Compartment Syndrome. The American journal of case reports. 2017;18:926-30.
48. Perera NJ, Steinbeck KS, Shackel N. The adverse health consequences of the use of multiple performance-enhancing substances--a deadly cocktail. The Journal of clinical endocrinology and metabolism. 2013;98(12):4613-8.
49. Perez-Downes J, Hritani A, Baldeo C, Antoun P. Amphetamine Containing Dietary Supplements and Acute Myocardial Infarction. Case reports in cardiology. 2016;2016 (no pagination)(6404856).
50. Peterson E, Stoebner A, Weatherill J, Kutscher E. Case of acute psychosis from herbal supplements. South Dakota medicine : the journal of the South Dakota State Medical Association. 2008;61(5):173-7.
51. Pourmand A, Li A, Yiu A, Mazer-Amirshahi M, Shokoohi H. Survival after profound acidosis and rhabdomyolysis due to dietary supplement use. American Journal of Emergency Medicine. 2016;34(11):2259.e1-.e3.
52. Pretegiani E, Rosini F, Rocchi R, Ginanneschi F, Vinciguerra C, Optican LM, et al. GABAAergic dysfunction in the olivary-cerebellar-brainstem network may cause eye oscillations and body tremor. Clinical neurophysiology : official journal of the International Federation of Clinical Neurophysiology. 2017;128(3):408-10.
53. Prosser JM, Majlesi N, Chan GM, Olsen D, Hoffman RS, Nelson LS. Adverse effects associated with arginine alpha-ketoglutarate containing supplements. Human & experimental toxicology. 2009;28(5):259-62.
54. Rhidian R. Running a risk? Sport supplement toxicity with ephedrine in an amateur marathon runner, with subsequent rhabdomyolysis. BMJ case reports. 2011;2011.
55. Ringer M, Pulfrey S. Runner's new diet, his collapse, and his ECG: when a rapid ECG diagnosis can save the day. Canadian family physician Medecin de famille canadien. 2014;60(4):340-4.
56. Ryan M, Lazar I, Nadasdy GM, Nadasdy T, Satoskar AA. Acute kidney injury and hyperbilirubinemia in a young male after ingestion of Tribulus terrestris. Clinical nephrology. 2015;83(3):177-83.
57. Saidi H, Mani M. Severe metabolic acidosis secondary to coadministration of creatine and metformin, a case report. The American journal of emergency medicine. 2010;28(3):388.e5-6.
58. Silverberg NB. Whey protein precipitating moderate to severe acne flares in 5 teenaged athletes. Cutis. 2012;90(2):70-2.
59. Simonart T. Acne and whey protein supplementation among bodybuilders. Dermatology (Basel, Switzerland). 2012;225(3):256-8.
60. Singh V, Rudraraju M, Carey EJ, Byrne TJ, Vargas HE, Williams JE, et al. Severe hepatotoxicity caused by a methasteron-containing performance-enhancing supplement. Journal of Clinical Gastroenterology. 2009;43(3):287.
61. Singhapricha T, Pomerleau AC. A Case of Strychnine Poisoning from a Southeast Asian Herbal Remedy. The Journal of emergency medicine. 2017;52(4):493-5.
62. Smedema JP, Muller GJ. Coronary spasm and thrombosis in a bodybuilder using a nutritional supplement containing synephrine, octopamine, tyramine and caffeine. South African Medical Journal. 2008;98(5):372-3.
63. Smith TB, Staub BA, Natarajan GM, Lasorda DM, Poornima IG. Acute myocardial infarction associated with dietary supplements containing 1,3-dimethylamylamine and Citrus aurantium. Texas Heart Institute journal. 2014;41(1):70-2.
64. Sousa MJCS, Ferreira ALR, da Silva JPM. Bodybuilding protein supplements and cow's milk allergy in adult. European Annals of Allergy and Clinical Immunology. 2018;50(1):42-4.
65. Thomas DB, Hall AB, Michel M. Non-cirrhotic hepatocellular carcinoma in a young active duty male. Military medicine. 2011;176(4):475-6.
66. Thomas JE, Munir JA, McIntyre PZ, Ferguson MA. STEMI in a 24-year-old man after use of a synephrine-containing dietary supplement: A case report and review of the literature. Texas Heart Institute journal. 2009;36(6):586-90.
67. Unnikrishnan D, Annam R, Jacob A, Thyagarajan B, Farrugia P. STEMI in a Young Male after Use of Synephrine-Containing Dietary Supplement. Case reports in cardiology. 2018;2018:7074104.
68. Velema MS, de Ronde W. Elevated plasma creatinine due to creatine ethyl ester use. The Netherlands journal of medicine. 2011;69(2):79-81.
69. Vilella AL, Limsuwat C, Williams DR, Seifert CF. Cholestatic jaundice as a result of combination designer supplement ingestion. Annals of Pharmacotherapy. 2013;47(7-8):e33.
70. Williamson L, New D. How the use of creatine supplements can elevate serum creatinine in the absence of underlying kidney pathology. BMJ case reports. 2014;2014.
71. Wong SS, Morrison-Reyes JA, Smithen LM. A case of central serous chorioretinopathy associated with use of deer antler spray supplement. Ophthalmic surgery, lasers & imaging retina. 2014;45(3):256-8.
72. Young C, Oladipo O, Frasier S, Putko R, Chronister S, Marovich M. Hemorrhagic stroke in young healthy male following use of sports supplement Jack3d. Military medicine. 2012;177(12):1450-4.
