# Supplementary material for: Sports nutrition supplements and adverse events – a meta-epidemiological study of case reports specifically addressing causality assessment
Source: Eur J Clin Pharmacol. 2021 Oct 2;78(1):1–9. doi: 10.1007/s00228-021-03223-9 (PMC8724217; doi:10.1007/s00228-021-03223-9)
Supplement: Supplementary file 3 — Supplementary file3 (DOCX 18 KB) [file 228_2021_3223_MOESM3_ESM.docx]

**Sports nutrition supplements and adverse events – a meta-epidemiological study of case reports specifically addressing causality assessment**

*Journal name: European Journal of Clinical Pharmacology*

Authors: Rickard Zeijlon, MD^1,2^, Victor Hantelius, MD^1^, Susanna M. Wallerstedt, MD, Professor^3,4^, Lina Holmqvist, MD, PhD^1,2^

^1^Department of Internal Medicine, Sahlgrenska University Hospital/S, Gothenburg, Sweden

^2^Department of Medicine, Sahlgrenska Academy, University of Gothenburg, Sweden

^3^Department of Pharmacology, Sahlgrenska Academy, University of Gothenburg, Sweden

^4^HTA Centre, Sahlgrenska University Hospital, Gothenburg, Sweden

E-mail of corresponding author:

rickard.zeijlon@gu.se

Online Resource 3. References for articles excluded after full text review.

1. Bento C, Velho P, Carvalho M. Lots of steroids and vitamins, tons of complications. Hypercalcemia and nephrocalcinosis as important complications of performance-enhancing drugs. Nefrologia : publicacion oficial de la Sociedad Espanola Nefrologia. 2015;35(6):598-600.

*Wrong intervention, not sports nutrition supplement*

1. Singh S, Gupta A, Sarkar S. Mephentermine Dependence in a Young Athlete: Case Report With Review of Literature. Journal of addiction medicine. 2017;11(4):328-30.

*Wrong intervention, not sports nutrition supplement*

1. Della Guardia L, Cavallaro M, Cena H. The risks of self-made diets: the case of an amateur bodybuilder. Journal of the International Society of Sports Nutrition. 2015;12:16.

*Wrong outcome, no specified adverse event*

1. Thomas A, Kohler M, Mester J, Geyer H, Schanzer W, Petrou M, et al. Identification of the growth-hormone-releasing peptide-2 (GHRP-2) in a nutritional supplement. Drug testing and analysis. 2010;2(3):144-8.

*Wrong outcome, no specified adverse event*

1. Hughes J, Shelton B, Hughes T. Suspected dietary supplement injuries in special operations soldiers. Journal of special operations medicine : a peer reviewed journal for SOF medical professionals. 2010;10(3):14-24.

*Wrong design, review of previous cases*
